# Efficacy of sorafenib adjuvant therapy in northwestern Chinese patients with non-metastatic renal-cell carcinoma after nephrectomy: A multicenter retrospective study

**DOI:** 10.1097/MD.0000000000014237

**Published:** 2019-02-01

**Authors:** Di Wei, Guojun Wu, Yu Zheng, Fubao Chen, Jingyi Lu, Yangmin Wang, Dalin He, He Wang, Zhiping Wang, Peng Chen, Yujie Wang, Zhiyong Wang, Yongli Ye, Zheng Zhu, Jianlin Yuan

**Affiliations:** a Department of Urology, Xijing Hospital, Fourth Military Medical University; b Department of Urology, General Hospital of Ningxia Medical University, Yinchuan, Ningxia Hui Autonomous Region; c Department of Urology, Xinjiang karamay Central Hospital, Karamay, Xinjiang Uyghur Autonomous Region; d Department of Urology, General Hospital of Lanzhou Military Area Command of Chinese PLA, Lanzhou, Gansu Province; e Department of Urology, The First Affiliated Hospital of Xi’an Jiaotong University; f Department of Urology, Tangdu Hospital, Fourth Military Medical University, Xi’an, Shaanxi Province; g Department of Urology, The Second Affiliated Hospital of Lanzhou University, Lanzhou, Gansu Province; h Department of Urology, Affiliated Tumor Hospital of Xinjang Medical University; i Department of Urology, The First Affiliated Hospital of Xinjiang Medical University, Urumqi, Xinjiang Uyghur Autonomous Region, China.

**Keywords:** adjuvant therapy, non-metastatic renal cell carcinoma, sorafenib

## Abstract

Recent studies have confirmed the efficacy of sorafenib for patients with advanced renal cell carcinoma; however, its efficacy and safety as an adjuvant therapy in patients with non-metastatic and loco-regional renal cell carcinoma after surgery remains controversial. Thus, the aim of the present retrospective study was to evaluate the efficacy of adjuvant sorafenib therapy in such patients from 8 centers in northwestern China that were treated from August 2009 to December 2016.

After surgery, the patients (n = 48) received oral sorafenib for 3 months. The control group (n = 48) comprised patients that underwent the same surgery from December 2009 to June 2016 but without adjuvant therapy who were matched 1:1 with the sorafenib group with respect to sex, age, pathological findings, disease stage and grade, operation time, and surgical procedure. The primary outcome compared between the groups was disease-free survival. Adverse events were also recorded to evaluate the safety of sorafenib. The influence of patients’ characteristics and laboratory tests on recurrence was analyzed using unconditional logistic regression.

Overall, the demographic characteristics of the 2 groups were similar. There was no significant difference in the rate of recurrence (8.3% for sorafenib patients and 6.2% for the matched patients, *P* = .66) or median disease-free survival between the 2 groups (hazard ratio = 1.561, 95% confidence interval = 0.349–6.987, *P* = .56). In multiple logistic regression analysis, increased blood urea nitrogen (BUN) emerged as an independent predictor of recurrence risk (*P* = .02).

These results indicate that postoperative sorafenib adjuvant therapy did not achieve the expected beneficial effect, pointing to the need for further studies to evaluate its utility in such cases.

## 1 Introduction

Renal cell carcinoma (RCC) is a cancer derived from the renal tubular epithelial cells, and is among the 10 most common cancers worldwide,^[1]^ with approximately 295,000 new diagnoses each year, accounting for approximately 2% of all new carcinoma patients. Approximately 134,000 people die of RCC annually, although the proportion of long-term survivors may be higher in developed countries.^[2,3]^ Some studies indicated that 20–30% of patients had already developed metastases at diagnosis, whereas those with localized RCC (∼16% of all cases) still have an approximately 40% recurrence rate after surgery.^[2,4,5]^ The recurrence risk for primary resected RCC can be estimated based on clinical and histological features in conjunction with TNM staging.^[6–8]^

The major pathological type of RCC is clear cell renal cell carcinoma (ccRCC), which is characterized by an inactive Von Hippel-Lidau (VHL) gene due to mutations and aberrant methylation, leading to abnormal overexpression of vascular endothelial growth factor (VEGF), platelet-derived growth factor (PDGF), and hypoxia-inducible factor (HIF)-1α and HIF2-α.^[9–12]^ These factors are all beneficial for tumor angiogenesis, and further lead to activation of the Raf/MEK/ERK pathway that promotes tumor cell survival and proliferation.^[13]^ Moreover, RCC pathogenesis has been related to immune system dysfunction. For example, loss of mature dendritic cells and the reduction of central memory T cells in lymphoid tissues surrounding the tumor can significantly suppress the anti-tumor function of T cells.^[14,15]^ In addition, certain gene mutations have been linked to RCC. For example, de Martino et al^[16]^ found that patients with *JAK3* mutations have a higher possibility of metastasis.

Based on these characteristics and its high rate of resistance to conventional chemotherapy,^[17]^ targeted tyrosine kinase inhibitor (TKI) are the first-line drugs for treatment of RCC. In particular, the TKI sorafenib is an oral multi-kinase inhibitor that mainly targets the VEGF and PDGF pathways, thereby suppressing tumor proliferation and angiogenesis,^[18]^ and shows potent anti-tumor activity in patients with metastatic RCC.^[19,20]^ Furthermore, sorafenib also showed a significantly greater therapeutic effect for RCC patients compared with interferon treatment.^[21]^

Although the efficacy of sorafenib has been extensively studied in patients with advanced-stage RCC, there are relatively few studies on its effectiveness as adjuvant therapy for early-stage RCC. Previous prospective studies, ASSURE and S-TRAC, explored these effects, but found different results. Based on these conflicting findings, we conducted the present retrospective analysis including patients from 8 centers in northwestern China that received sorafenib treatment and matched controls without adjuvant treatment post-surgery.

## 2 Methods

### 2.1 Study design

This multicenter retrospective study was conducted using a matched-pairs design with a 1:1 ratio between sorafenib and control patients. The sorafenib patients received the drug postoperatively via oral administration. The matching criteria were based on pathological examination, TNM stage, Fuhrman grade, sex, age, operation time, and surgical procedure. If the patients could not be completely matched, we appropriately broadened the matching criteria and chose the most similar patient as the paired control.

### 2.2 Patients and treatments

From August 2009 to December 2016, we collected the data of 96 patients that underwent tumor resection for localized RCC from 8 centers in northwestern China, with 48 patients each in the sorafenib and matched non-sorafenib group. All patients were pathologically diagnosed with RCC, and were >18 years of age. Other inclusion criteria included: no significant liver and kidney function damage (Child-Pugh score C or above, creatinine clearance <30 mL/min), no second tumor within 5 years, no major cardiovascular events within 6 months prior to treatment, no severe uncontrolled blood pressure (>150/100 mmHg). None of the patients received any systemic anti-tumor therapy. All of the patients were supported by ethics committee of Xijing Hospital.

The patients in the sorafenib group received 400 mg of sorafenib twice daily for 3 months continuously after the operation. Adverse events were monitored every month during the treatment. Within 3 months of the start of treatment, the patients were followed up once a month, which was subsequently changed to once every 6 months. Tumor recurrence, metastasis, or the presence of new tumors was evaluated by imaging examinations (computed tomography or magnetic resonance).

### 2.3 Safety assessment

The safety evaluation of sorafenib included adverse events, laboratory tests, score on the Eastern Cooperative Oncology Group (ECOG) scale (from 0 to 5, with higher scores indicating greater disability), and 12-lead echocardiogram. The assessment of adverse events included the type, duration, and grade, according to the Common Terminology Criteria for Adverse Events version 3.0 (CTCAE v3.0).

### 2.4 Statistical analysis

Disease-free survival (DFS) was the main outcome measure used for comparison between the groups, which was defined as the duration from surgery until tumor recurrence, and was visually assessed with a Kaplan–Meier plot. Continuous data are presented as means ± standard deviations, and count data are represented by the number of cases and their percentages. Continuous data were compared using independent *t* tests, and the count or categorized data were compared using the chi-squared test. The influence of patients’ characteristics and laboratory variables on recurrence was analyzed by unconditional logistic regression. Statistical significance (*P* < .05) was analyzed using SPSS 21.0 (IBM Corporation, USA).

## 3 Results

### 3.1 Patient characteristics

The basic clinical features of the patients are listed in Table 1. Among the 96 patients, the average age of the sorafenib group and matched group did not differ significantly (50.08 ± 11.37 and 51.16 ± 11.45 years, respectively). The distribution of ethnicity was also similar between the groups, with 45 and 46 patients of Han ethnicity and 3 and 2 patients of Uyghur ethnicity in the sorafenib and matched group, respectively. The follow-up period for the sorafenib group was August 2009 to April 2018, whereas that of the matched group was December 2009 to December 2017. The patients’ risk stage was assessed based on the University of California, Los Angeles Integrated Staging System (UISS). The age, sex, history, surgery, AJCC stage, pathological TNM stage, Furman nuclear grade, ECOG score, and UISS risk stage did not differ significantly between the groups (Table 1).

**Table 1:**
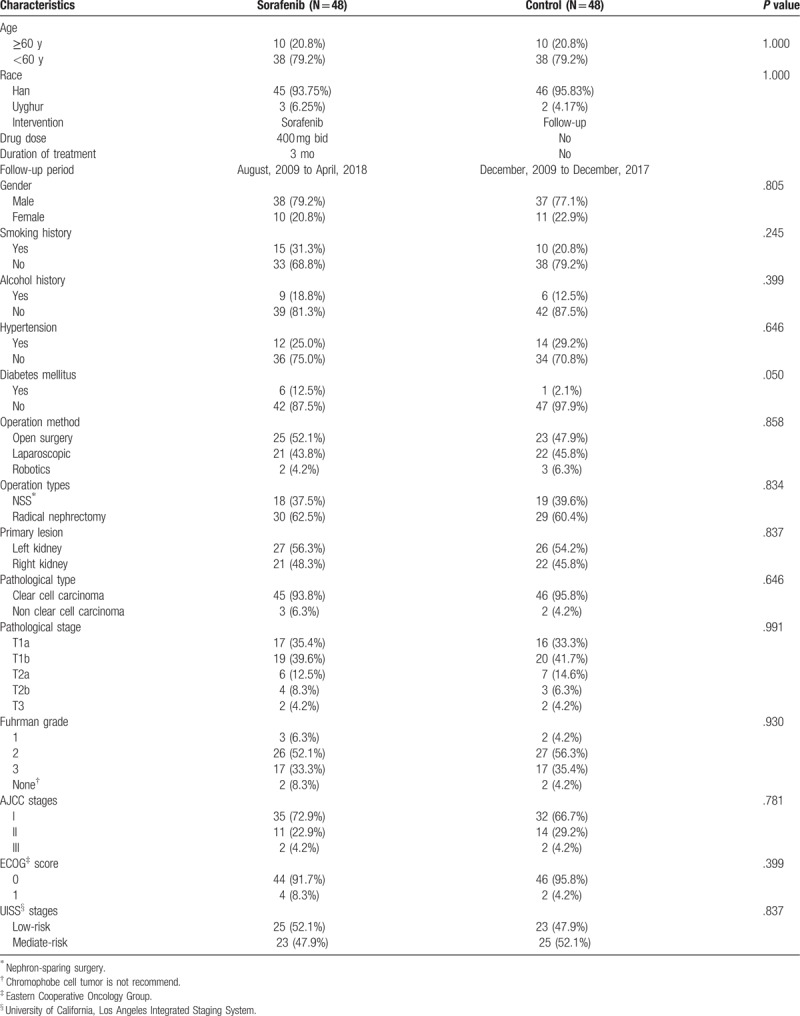
Basic characteristics of the patients.

In the sorafenib group, there were 38 male and 10 female patients. According to the UISS, 25 and 23 patients were classified as low and intermediate risk, respectively. In the matched group, there were 37 male and 11 female patients, with 23 and 25 classified as low and intermediate risk, respectively.

The matched patients were followed up with no further treatment, whereas the patients in the sorafenib group received 400 mg of sorafenib twice daily within 4 to 12 weeks after surgery, and continued the therapy for 3 months. No patients had to discontinue treatment owing to adverse events.

### 3.2 Treatment efficacy

**Figure 1:**
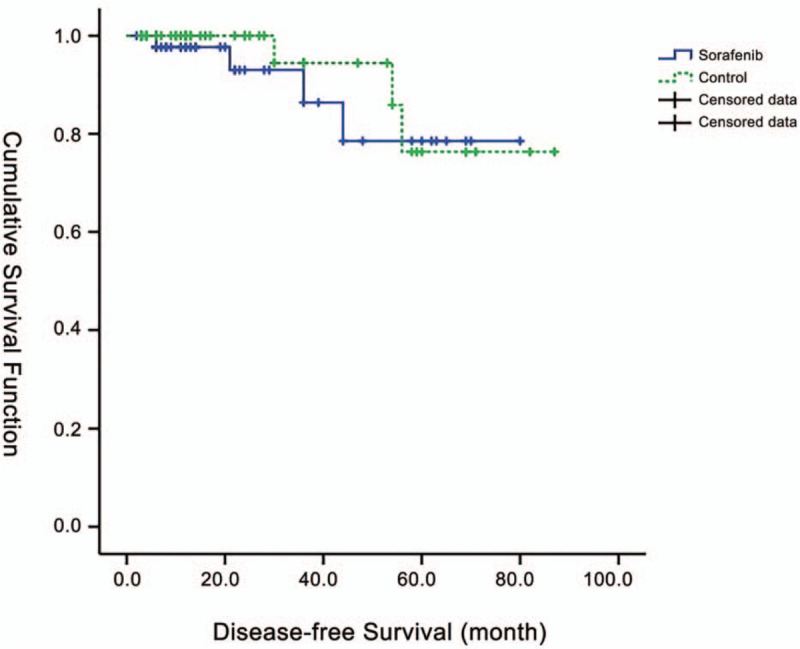
Kaplan–Meier plot of cumulative disease-free survival (DFS).

Among the sorafenib-treated patients, the overall rate of recurrence was 8.3% (4/48), with liver, lymph node, lung, and bone metastasis in 1 patient each. The overall rate of recurrence in the matched patients was 6.2% (3/48), with liver, bone, and lung metastasis in 1 patient each (Table 2). The median DFS for RCC patients was not reached in either group, and the risk of recurrence was not significantly different between groups (hazard ratio = 1.561, 95% confidence interval = 0.349–6.987, *P* = .557) (Fig. 1).

**Table 2:**
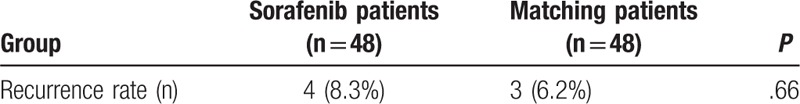
Efficacy of sorafenib patients and matched patients in preventing recurrence.

### 3.3 Safety

A total of 21 different types of adverse events were recorded in the sorafenib patients, including at least one adverse event in every patient (Table 3). The most common adverse events were hand-foot syndrome, alopecia, rash, diarrhea, hypertension, and cutaneous pruritus. Grade-3 adverse events were reported in 15 patients. One or two types of adverse events occurred in 20 patients, and 3 or 4 types occurred in 11 patients. There were 17 patients with >5 adverse events. One patient experienced 10 different types of adverse events. Nevertheless, the treatment did not need to be and the drug dose did not have to be reduced due to adverse events for any patient.

**Table 3:**
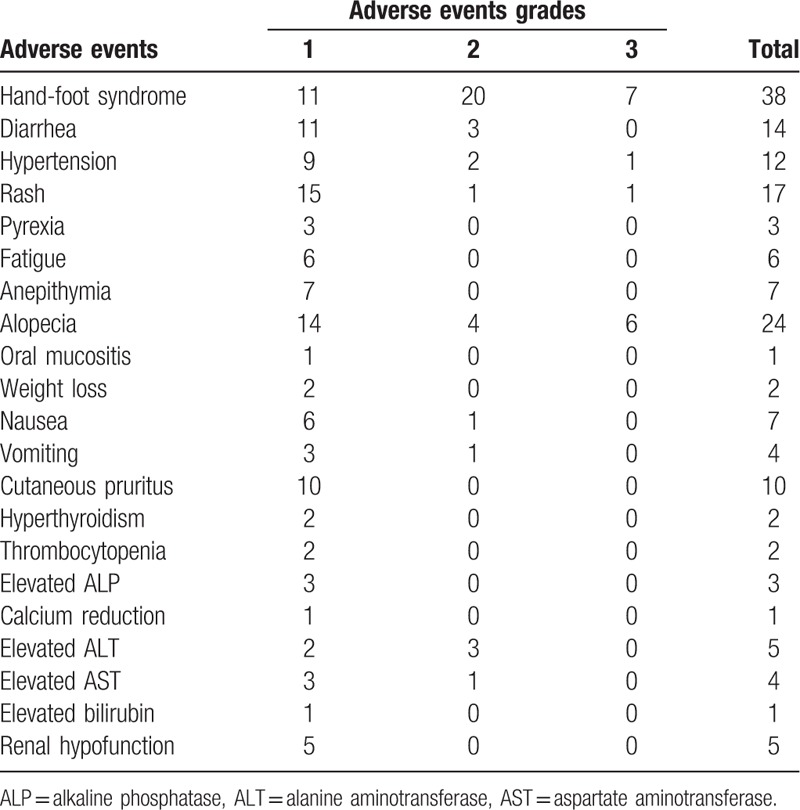
Adverse events in sorafenib-treated patients.

### 3.4 Recurrence risk factors

The patients that experienced recurrence had increased levels of blood urea nitrogen (BUN) (*χ*^2^ = 4.593, *P* = .03) and serum creatinine (Scr) (*χ*^2^ = 5.761, *P* = .02) (Table 4). However, univariate non-conditional logistic regression analysis demonstrated that age (*P* = .01), hypertension (*P* = .02), UISS risk group (*P* = .03), Fuhrman grade (*P* = .02), decreased alkaline phosphatase (ALP) (*P* = .03), increased total bilirubin (TBI) (*P* = .04), increased BUN (*P* = .01), and increased Scr (*P* = .01) may be potential recurrence risk factors (Table 5). However, after removing the confounding factors and including these indices in multiple logistic regression analysis, only BUN remained a significant independent predictor of recurrence risk (*P* = .02) (Table 6).

**Table 4:**
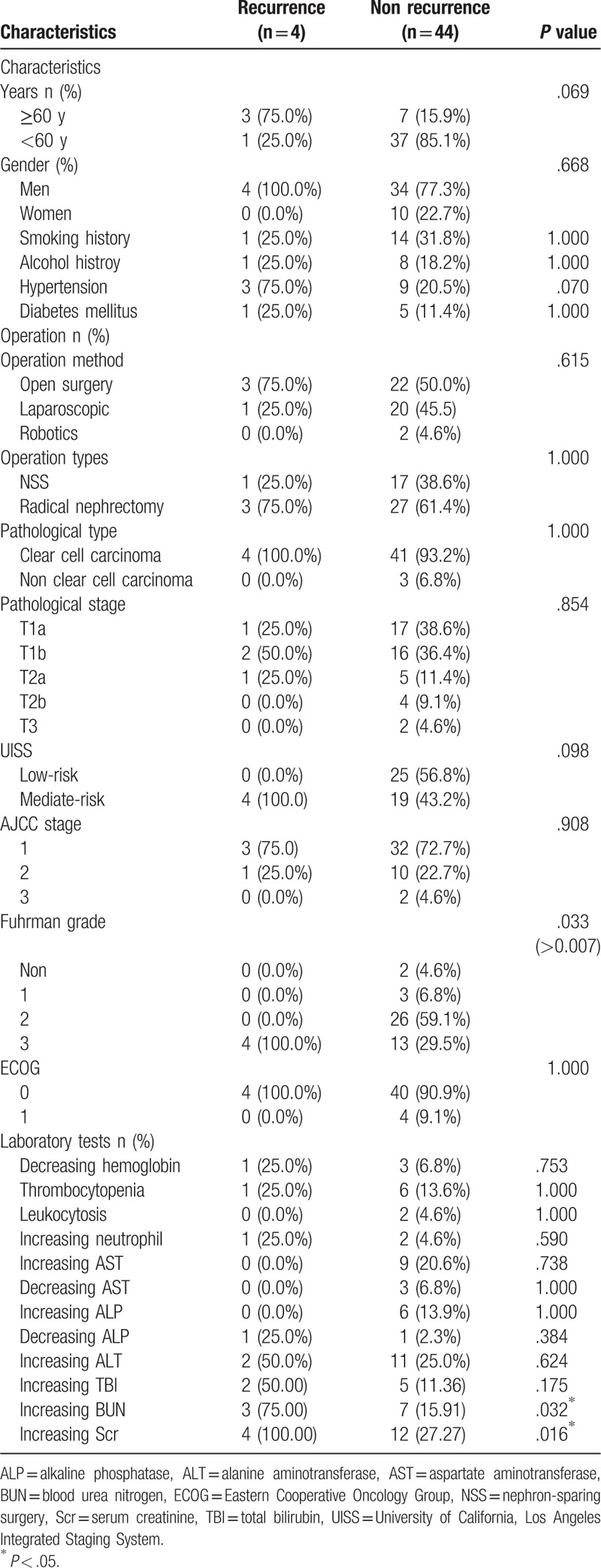
Clinical characteristics and laboratory tests of the sorafenib-treated patients in relation to recurrence (chi-squared test).

**Table 5:**
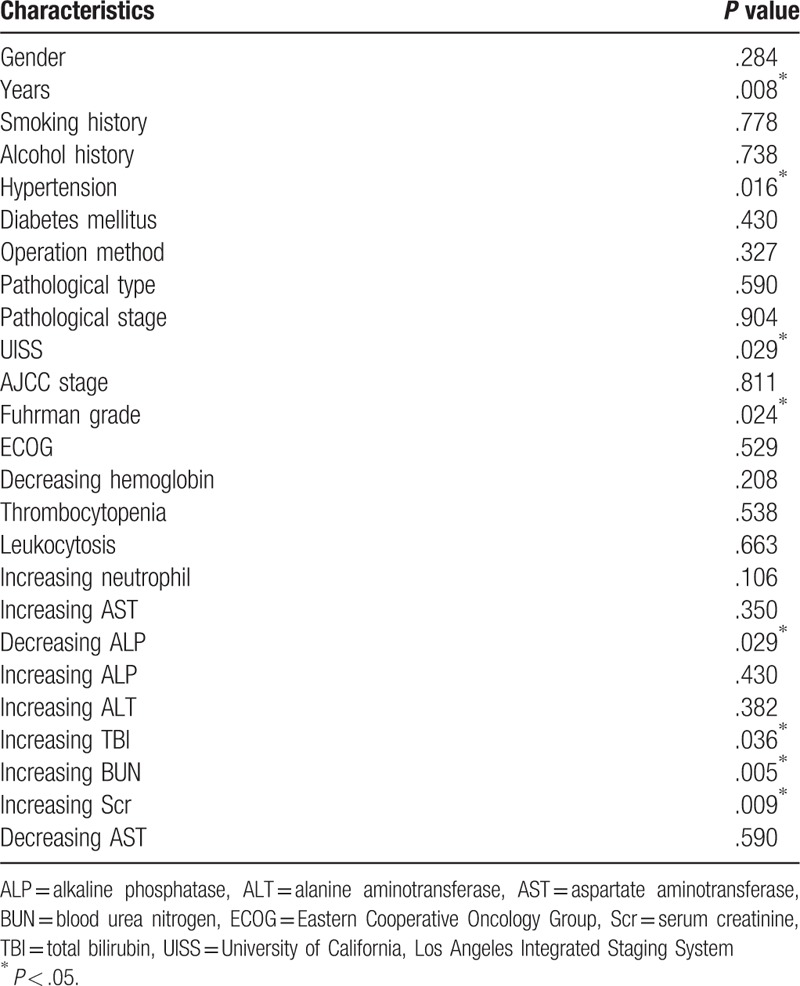
Univariate non-conditional logistic regression analysis of the sorafenib patients’ clinical characteristics and laboratory tests in relation to recurrence.

**Table 6:**
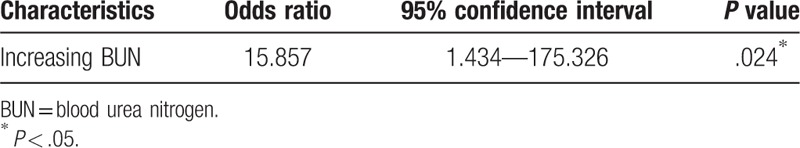
Multivariate non-conditional logistic regression analysis of sorafenib patients’ clinical characteristics and laboratory tests in relation to recurrence.

## 4 Discussion

Surgery and medicine are the main treatment options available for patients with RCC. However, with increasing recognition of the importance of the immune system in tumor biology, tumor vaccines are emerging as a new treatment method in several types of cancers, including RCC. Gigante et al^[15]^ isolated and cultured dendritic cells from patients with RCC, and was able to successfully activate cytotoxic lymphocytes, which contributed to the development of tumor vaccines. However, the current effective treatment after surgery remains targeted drugs.

Previous studies have shown that TKIs have significant therapeutic efficacy in patients with advanced RCC^[22]^; however, in patients with early, local RCC, the application of sorafenib as an adjuvant therapy after surgery is controversial. The main representative clinical trials on TKI adjuvant therapy in RCC are ASSURE, and S-TRAC. In the ASSURE trial conducted by Haas et al,^[23]^ the median DFS did not differ significantly between the sorafenib-treated patients and placebo group (6.1 vs 6.6 years). However, the opposite result was obtained in the S-TRAC trial, showing a median DFS of 6.8 years and 5.6 years in the sunitinib and placebo group, respectively.^[24]^ Following assessment of an independent blinded central review board, sunitinib adjuvant therapy was concluded to be superior to placebo.

This is the first study to investigate the efficacy and safety of a TKI as an adjuvant treatment for early and local RCC in a northwestern Chinese population. The main pathological type in this cohort was ccRCC. Since sorafenib targets VEGF and PDGF, this group was appropriate for evaluation of the effect of TKIs. In addition, the study population was distributed across the major provinces of northwestern China, making it a geographically representative sample. Similar to the ASSURE trial, there were no significant differences in the survival time between the sorafenib-treated patients and matched controls, and the median DFS was not reached in either group.

In general, 40% of patients with RCC can now survive for 10 years after surgery,^[25]^ which is largely attributed to advances in surgical techniques. In northwestern China, the administration of sorafenib adjuvant therapy in patients with RCC was mainly only started in 2011. Therefore, in this retrospective study, we did not reach the median DFS. However, analysis of the overall survival time still showed no difference between the groups. Hence if we get the media DFS, the results may be different.

Thus, our results provide further support for no benefit of sorafenib as adjuvant treatment after surgery in early-stage RCC patients; thus, the controversy remains, and is worthy of further study. The different results obtained among studies may be related to variations in the distribution of pathological type and tumor risk stage in the patients in each trial. In the ASSURE trial, 79% of the patients had ccRCC, and 33% were classified in AJCC stage 1–2. In the S-TRAC trial, 99% of the patients also had ccRCC; however, they were all at AJCC stage 3 or higher. Similarly, in the ASSURE trial, the mid-high risk was based on T1, Fuhrman grade 3–4; T2, T3, Fuhrman grade 1; T3, Fuhrman grade ≥ 1; and the ECOG score was 0, whereas in the S-TRAC trial, all patients were at T3 or higher. Staehler et al^[26]^ reported that in the S-TRAC trial, the higher-risk patients (i.e., higher recurrence and metastasis rates) were those that received a benefit from sorafenib. Haas et al^[27]^ reanalyzed the results of the ASSURE trial by selecting only patients with ccRCCs who were at least at middle risk, and found no significant difference in the 5-year PFS between the sorafenib and placebo groups, further contributing to the controversy of the benefit of postoperative adjuvant therapy. In our study, 95% of the patients were at AJCC stage 1–2 and there were far fewer patients at stage T3 compared with those in the ASSURE trial. Hence, this may have contributed to the difference in the results between trials. In contrast to the ASSURE and S-TRAC trial, the patients received sorafenib for 1 year, whereas our patients only received sorafenib for 3 months; thus, it is possible that sustained therapy will have some benefits down the road. However, we do not recommend a longer course owing to the financial burden for the patients and adverse effects of the drug. Moreover, we hypothesize that immune system dysfunction plays a dominant role in the development of RCC; however, the targeted drug in this case cannot affect the patient's immune system. Hence, adjuvant therapy may have a limited benefit for patients with early-stage RCC.

According to the results of the chi-squared test, the frequencies of increased BUN and increased Scr were higher in the patients that experienced recurrence. However, this does not necessarily mean that these factors are the cause of the recurrence. Univariate non-conditional logistic regression analysis showed many factors that could affect recurrence, including older age, hypertension, UISS risk group, Fuhrman grade, decreased ALP, increased TBI, increased BUN, and increased Scr. It is generally believed that the human organ function gradually declines with age, leading to an increased risk of tumorigenesis in older individuals. Indeed, a previous study showed that older female RCC patients had a worse prognosis.^[28]^ The UISS risk group and Fuhrman grade are not only used to evaluate the RCC grade but also serve as prognostic predictors. TBI, Scr, and BUN, can reflect renal function, and thus their abnormal levels often raises alarm, leading to closer monitoring. However, our multiple logistic regression analysis demonstrated that only increased BUN can be used as a predictor of recurrence risk. Nevertheless, this result may have been influenced by the relatively small sample size; hence, further data with larger populations will be needed to verify this association.

The most common adverse events were hand-foot syndrome, alopecia, rash, diarrhea, hypertension, and cutaneous pruritus, which is consistent with previous studies on sorafenib.^[16,20]^ Nevertheless, the dose of sorafenib did not need to be reduced and the drug did not need to be discontinued in any patient due to unacceptable adverse events. This may be related to the fact that our patients only took sorafenib for 3 months after surgery, which is a relatively short time.

## 5 Conclusion

In this first study of the effects of sorafenib as postoperative adjuvant therapy for RCC in a northwestern Chinese population, we did not observe a beneficial therapeutic effect, contributing to the ongoing controversy. Thus, continued follow-up and inclusion of new patients in this study will help to supplement these results and hopefully provide resolution to the controversy or explanation as to why these effects differ from those for patients at more advanced disease stages.

## Acknowledgments

The authors are grateful to the clinicians and staffs at the 8 clinical centers in northwestern China who contributed to the samples and data collection for this study.

## Author contributions

**Conceptualization:** Di Wei, Guojun Wu, Yu Zheng, Jianlin Yuan.

**Data curation:** Di Wei, Fubao Chen, Jingyi Lu, Yangmin Wang, Dalin He, He Wang, Zhiping Wang, Peng Chen, Yujie Wang, Zhiyong Wang, Yongli Ye, Zheng Zhu.

**Formal analysis:** Di Wei, Yu Zheng.

**Supervision:** Guojun Wu, Jianlin Yuan.

**Visualization:** Guojun Wu.

**Writing – original draft:** Di Wei.

**Writing – review & editing:** Jianlin Yuan.
